# A new species of *Dendropsophus* (Anura, Hylidae) of the *D. ruschii* group from the Atlantic Forest in Serra da Mantiqueira, Minas Gerais, Brazil

**DOI:** 10.1371/journal.pone.0351087

**Published:** 2026-06-23

**Authors:** Diego J. Santana, Donald B. Shepard, Priscila S. Carvalho, Márcia M. P. Müller, Clodoaldo L. Assis, Renato N. Feio

**Affiliations:** 1 Negaunee Integrative Research Center and Keller Science Action Center, Field Museum of Natural History, Chicago, Illinois, United States of America; 2 Department of Biological Sciences, University of Arkansas, Fayetteville, Arkansas, United States of America; 3 Instituto de Biociências, Letras e Ciências Exatas, Universidade Estadual Paulista “Júlio de Mesquita Filho”, São José do Rio Preto, São Paulo, Brazil; 4 Museu de Zoologia João Moojen, Departamento de Biologia Animal, Universidade Federal de Viçosa, Viçosa, Minas Gerais, Brazil; Universidade Estadual Paulista: Universidade Estadual Paulista Julio de Mesquita Filho, BRAZIL

## Abstract

The *Dendropsophus ruschii* species group currently comprises two species with disjunct distributions between the Atlantic Forest and the Amazon. Based on an integrative approach combining morphological, acoustic, and molecular data (mtDNA barcoding), we describe a new species from the Serra da Mantiqueira, Minas Gerais, Brazil. *Dendropsophus liliae* sp. nov. is diagnosed by its small size, rounded digital discs, presence of a calcar appendage, dark red iris, and a distinct white stripe from the snout to the upper eyelid. This discovery expands the known diversity of the group and represents its most inland record within the Atlantic Forest.

Historical connections between Atlantic Forest and Amazon are evident from their many shared biotic similarities [1]. Furthermore, the evolutionary histories of diverse animal groups have been shaped by recurrent connections between these forested ecoregions with many lineages occurring in both forests such as birds [2,3], reptiles [4–8], and amphibians [9–12]. Despite many amphibian groups sharing evolutionary histories among Atlantic Forest and Amazon, there is not a single species occurring in both ecoregions [9,12,13]. Frogs of the genus *Adelophryne* (Eleutherodactylidae, Phyzelaphryninae) and *Allophryne* (Allophrynidae) are prime examples of taxa with closely related species exclusive to either Atlantic Forest or Amazon [9,14].

Recent phylogenies and systematic reviews of anurans have revealed new evolutionary hypotheses, which were paramount for better organizing anuran taxonomy [15–17]. Among the recent revisions of neotropical anurans, the tribe Dendropsophini Faivovich, Haddad, Garcia, Frost, Campbell, and Wheeler, 2005 has undergone a comprehensive systematic and taxonomic review [18], which rearranged species groups. Although recent phylogenomic analyses have suggested that the *D. ruschii* group may be paraphyletic, with the transfer of *D. ruschii* to the *D. decipiens* group and *D. ozzyi* remaining *incertae sedis* [19], we follow the species group arrangement proposed by Orrico et al. [18], as it represents a focused systematic revision of the group and provides a consistent framework for comparison. Among the new arrangements, the *Dendropsophus ruschii* species group is composed of only two species: *D. ruschii* (Weygoldt and Peixoto, 1987) occurs in a few locations within Atlantic Forest in the states of Espírito Santo and Minas Gerais [20], while the recently described *D. ozzyi* Orrico, Peloso, Sturaro, Silva, Neckel-Oliveira, Gordo, Faivovich, and Haddad, 2014 occurs in the western Amazon. These two species share no obvious phenotypic similarities [18], except for calls composed of short, trilled notes with 3–5 pulses each (no secondary notes) and small size (SVL 18.5–27.9 mm for males and 26.7–29.0 mm for females) [21,22].

During recent field expeditions to Serra do Brigadeiro, in the north section of the Serra da Mantiqueira Mountain Range, a well-known center of endemism and diversity for Atlantic Forest anurans [23,24], we collected several specimens of *Dendropsophus* belonging to the *D. ruschii* species group. The distinct phenotype of these frogs, combined with their notable inland occurrence within the Atlantic Forest, prompted further investigation into their taxonomic identity. Here, we combine morphological, acoustic, and mtDNA barcoding evidence to demonstrate that these populations represent a lineage previously unknown to science, and we describe it as a new species.

## Materials and Methods

### Sampling

We conducted visual and acoustic surveys at Serra do Brigadeiro, Ervália municipality, Minas Gerais state, Brazil, in November 2021 and November 2022. All specimens were captured manually, killed using 5% lidocaine, had tissue samples (liver) taken, were fixed in 10% formalin, and transferred to 70% ethanol for permanent storage (following Conselho Federal de Biologia-CFBio Nº 148/2012). Voucher specimens are housed in the Museu de Zoologia João Moojen (MZUFV), Viçosa, Brazil, Coleção Zoológica da Universidade Federal de Mato Grosso do Sul (ZUFMS-AMP), Campo Grande, Brazil, and Museu Nacional do Rio de Janeiro (MNRJ), Rio de Janeiro, Brazil (Appendix I). Collections were authorized by the Instituto Chico Mendes de Conservação da Biodiversidade (ICMBio; SISBIO license #10504−4), and protocols for collection and handling of individuals used in this research were in accordance with Brazilian federal law.

### Morphology

Specimens used in the description of the new species, as well as specimens examined for comparison, are housed in four herpetological collections in Brazil (Appendix I). Terminology for morphological characters follows Orrico et al. [22]. We follow Duellman [25] for the eleven morphometric variables: SVL (snout-vent length), HL (head length), HW (head width), ED (eye diameter), TD (tympanum diameter), IOD (interorbital distance), END (eye-nostril distance), ELW (eyelid width), THL (thigh length), TL (tibia length), and FL (foot length). All measurements were taken by PSC using a digital caliper (0.01 mm precision). We determined the sex of each individual by the presence of vocal sacs and vocal slits in males and their absence in females. For web formulas, we follow Savage and Heyer [26,27].

### Bioacoustics

We recorded advertisement calls from one paratype (MZUFV20679) at the type locality and analyzed a total of 21 calls. Recordings were obtained using a Tascam DR-44 digital recorder around 20:00 h (air temperature 15 °C, water temperature 16 °C), with a sampling rate of 44,100 Hz and 16-bit resolution in WAV format. Acoustic analyses were performed in Raven Pro v1.5 for Mac [28], and audio spectrograms were generated in R using the package *seewave* [29] with the following settings: FFT window width = 256, frame = 100, overlap = 75, and flat-top filter. We measured the following acoustic parameters: call duration, and dominant frequency. Terminology and criteria for call descriptions follow Köhler et al. [30], in which a call is defined as an acoustic unit separated from other calls by silent intervals longer than the call itself (call-centered approach). Acoustic parameters are reported as mean ± SD (minimum–maximum). Sound recordings were deposited in the acoustic collection of the Fonoteca Mapinguari at Universidade Federal de Mato Grosso do Sul (MAP-V 331).

### Phylogenetic inference and genetic distances

Genomic DNA from two individuals was extracted from tissue samples using the QIAGEN DNeasy Blood and Tissue Kit (Valencia, California, USA), following the manufacturer’s instructions. We amplified a fragment of the mitochondrial 16S rRNA gene using primers 16Sar and 16Sbr [31]. PCR reactions consisted of an initial denaturation step at 94 °C for 3 min, followed by 35 cycles of 94 °C for 20 s, 50 °C for 20 s, and 72 °C for 40 s, with a final extension at 72 °C for 5 min. PCR purification and bidirectional sequencing were carried out by Eurofins Genomics Inc. (Louisville, Kentucky, USA).

We assembled a supermatrix by combining newly generated 16S sequences (PZ305670, PZ305671) with the complete dataset of Orrico et al. [18], including outgroup taxa. The final dataset comprised 5,802 base pairs and 216 terminals. Data partitioning was evaluated by gene (12S 992 bp; 16S 1,648 bp; COI 645 bp; Cytb 400 bp; POMC 444 bp; RAG-1 316 bp; SIA 397 bp; Tyr 532 bp), with protein-coding genes further subdivided by codon position (1st, 2nd, and 3rd). We selected the best-fitting partitioning scheme and nucleotide substitution models using PartitionFinder 2 under the Bayesian information criterion (BIC) [32] (Table 1).

**Table 1:** Best-fitting partitioning scheme model of nucleotide substitution for all genes used in the study.

Subset	Best Model	Base pairs	Partition names
1	GTR + I + G	1125	CYTB_pos2, 12S
2	GTR + I + G	1643	16S
3	TRN + G	215	COI_pos1
4	TRN + I + G	215	COI_pos2
5	HKY + I	320	RHO_pos3, COI_pos3
6	GTR + G	134	CYTB_pos1
7	K80 + I + G	265	CYTB_pos3, SIA_pos2
8	HKY + I + G	755	POMC_pos1, POMC_pos2, TYR_pos3, TYR_pos2, RHO_pos2
9	HKY + G	469	RAG1_pos2, POMC_pos3, TYR_pos1
10	K80 + I	417	SIA_pos3, RAG1_pos1, RAG1_pos3
11	K80 + G	239	RHO_pos1, SIA_pos1

Sequence alignments were generated using the MAFFT algorithm [33] implemented in Geneious v9.0.5 with default parameters. GenBank accession numbers and genetic voucher information are provided in Appendix S3 of Orrico et al. [18]. Phylogenetic relationships were inferred using Bayesian inference in BEAST v2.6.6 [34], with analyses run for 100 million generations, sampling every 10,000 generations, under a Yule speciation prior and a strict molecular clock. Convergence and stationarity were assessed by visual inspection of trace plots and by confirming effective sample sizes greater than 200 in Tracer v1.7.1 [35]. The first 10% of trees were discarded as burn-in, and a maximum clade credibility tree with median node ages was generated using TreeAnnotator v2.6.3 [34]. Pairwise genetic distances (uncorrected p-distances) among individuals of the *Dendropsophus ruschii* group were calculated in MEGA v10.1.1 [36].

### Nomenclatural acts

The electronic version of this article meets the requirements of the amended International Code of Zoological Nomenclature, and therefore the new names proposed herein are available under that Code. This work and the nomenclatural acts it includes have been registered in ZooBank, the official online registry of the ICZN. The Life Science Identifier (LSID) for this publication is: urn:lsid:zoobank.org:pub:D2A4DAB1-CE7A-4D38-A96D-79A49642AA0E. The electronic edition of this work was published in a journal with an ISSN, and has been archived and is available from the following digital repositories: CLOCKSS.

### Species Description

*Dendropsophus liliae* **sp. nov.**

(Figs 1–3)

**Fig 1:**
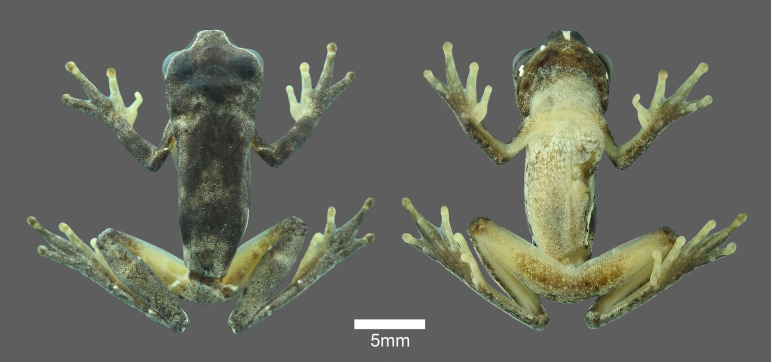
Holotype of *Dendropsophus liliae* sp. nov. (MZUFV20707). (A) Dorsal view of the body; B) ventral view of the body. Photographs by D.J. Santana. Published under a CC BY 4.0 license.

**Fig 2:**
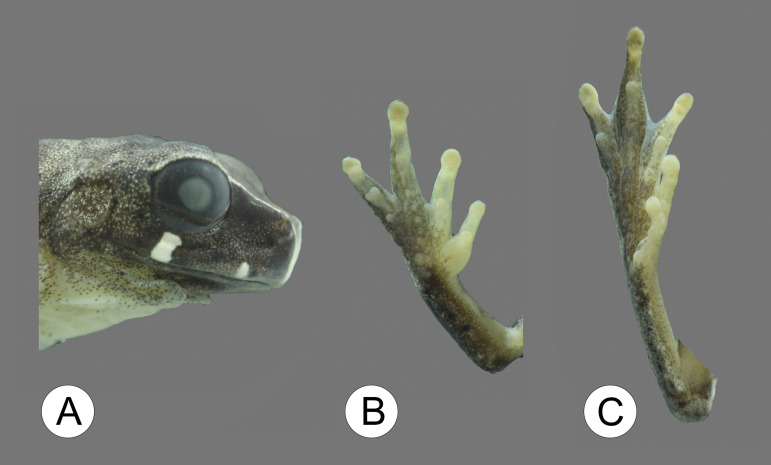
Holotype of *Dendropsophus liliae* sp. nov. (MZUFV20707). (A) Head lateral view; (B) ventral view of right hand; and (C) ventral view of right foot. Photographs by D.J. Santana. Published under a CC BY 4.0 license.

**Fig 3:**
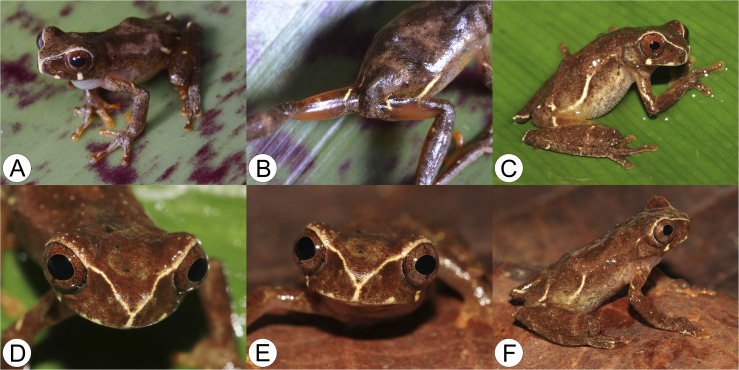
Live specimens of *Dendropsophus liliae* sp. nov. from Serra do Brigadeiro (type locality). (A) Holotype adult male (MZUFV20707), (B) paratype adult male (ZUFMS-AMP7816), (C–F) unvouchered adult males. Photographs by D.J. Santana (A–B) and C.L. Assis (C–F). Published under a CC BY 4.0 license.

*Dendropsophus ruschii* (Cassini et al. 2007 and Silva et al. 2018, in part)

LSID urn:lsid:zoobank.org:act:891C0168-DDBD-4B8A-9374-A8743885ACB0

**Holotype.—**MZUFV20707, adult male, from Serra do Brigadeiro, swamp in the trail to the Lagoa das Bromélias, Careço district, Ervália municipality, Minas Gerais state, Brazil (20º53’17”S, 42º31’43”W, ~ 1200 m above sea level (a.s.l.); datum = SAD69), collected on 4 November 2021, by Santana, D.J., Carvalho, P.S., Müller, M.M.P., Oliveira, H., Yves, A., Costa, H.C. and Feio, R.N.

**Paratypes.—**ZUFMS-AMP7816, MZUFV20458–20459, all adult males collected on 4 November 2021, by Santana, D.J., Carvalho, P.S., Müller, M.M.P., Oliveira, H., Yves, A., Costa, H.C. and Feio, R.N. in the same locality as the holotype; MZUFV20690–20697, MZUFV20679–20681, MNRJ94194–94196 (ex-MZUFV20703–20705), all adult males collected on 9 November 2022, by Assis, C.L. and Feio, R.N. in the same locality as the holotype.

**Diagnosis.—**We assigned the new species to the genus *Dendropsophus* and to the *D. ruschii* species group based on phylogenetic results (see Results below). In addition, the new species morphologically resembles other species of the *D. ruschii* group, especially *D. ruschii*, which exhibit white marks on the head and flanks. *Dendropsophus liliae* sp. nov. can be distinguished from its congeners within the *D. ruschii* group by the combination of the following features: (1) small size, adult males 16.0–19.5 mm SVL; (2) dominant frequency of the advertisement call from 6030 to 6630 Hz; (3) rounded discs on fingers and toes; (4) presence of sparse small granules on the dorsum; (5) presence of a calcar appendage; (6) eyes dark red; (7) overall dorsal coloration brown; (8) nuptial pad poorly developed; (9) wide head HW/SVL = 0.30–0.34; (10) a well-marked white line running from the posterior edge of the upper eyelid to the tip of the snout.

**Comparisons with other species (character in species under comparison in parentheses).—***Dendropsophus liliae* sp. nov. differs from *D. ozzyi* by the rounded discs on fingers and toes (pointed discs on fingers and toes); by the presence of sparse small granules on the dorsum (dorsum smooth); by the presence of a calcar appendage (calcar with no appendage); by dark red eye coloration (light cream eye coloration); by the overall dorsal coloration brown with white stripes from the tip of the snout to the posterior edge of the upper eyelid, below the eyes, and on the flanks (overall dorsal coloration cream to beige with sparse irregular stains); by the dominant frequency of the advertisement call from 6030 to 6630 Hz (dominant frequency in the advertisement call from 9130 to 10140 Hz).

*Dendropsophus liliae* sp. nov. differs from *D. ruschii* in SVL from 16.0 to 19.5 mm in adult males (adult males measuring from 24.5 to 27.9 mm); by nuptial pad poorly developed (nuptial pad well developed); by a wide head HW/SVL = 0.30–0.34 (HW/SVL = 0.28–0.30); by well-marked white line from the posterior edge of the upper eyelid to the tip of the snout (white line faded or absent from the upper eyelid to the tip of the snout); by the dominant frequency of the advertisement call from 6030 to 6630 Hz (dominant frequency in advertisement call from 3500 to 4500 Hz).

**Description of the holotype.—**Adult male as indicated by presence of vocal slits and large vocal sac; SVL 17.63 mm; body slender; head wider than body, head as long as wide (HW/HL = 1.00); snout short, truncate in dorsal and lateral views; distance from nostril to eye less than diameter of eye (END = 1.29 mm), ratio END/ED = 0.71–0.93; canthus rostralis distinct, rounded in lateral view; loreal region slightly concave, sloping to upper lip; nostrils barely protuberant, openings directed laterally; eye quite large (ED = 2.15 mm); tympanic membrane round, indistinct, visible, with diameter more than half of eye length (TD/ED = 0.58); tympanic annulus visible; supratympanic fold present covering the upper part of the tympanic annulus; forelimbs not hypertrophied; abbreviated axillary membrane discrete; minute ulnar tubercles absent; finger discs round, wider than digit; relative length of fingers I < II < IV < III; webbing vestigial between Fingers I and II, basal between Fingers II to IV; finger webbing formula I 2.5–3 II 2 + – 3 III 2⅔ –2.5 IV; distal subarticular tubercle on fourth finger flat; presence of few supernumerary tubercles; presence of two elliptical palmar tubercles; subarticular tubercle large, rounded; nuptial excrescences present; hind limbs long, slender, with calcar tubercles, tarsal fold small and discrete, formed by a sequence of small tubercles; toes moderately long, toe discs round; relative length of toes I < II < III < IV < V; outer metatarsal tubercle inconspicuous; inner metatarsal tubercle present, round; subarticular tubercles small, rounded; supernumerary tubercles present; toe webbing formula I 2–2.5 II 1.5–3 III 1.75–3 IV 2 + –1.5 V; dorsal surfaces of the eyelids with small, scattered tubercles; dorsum with scattered tubercles; skin on belly coarsely granular; cloacal opening directed posteriorly at upper level of thighs; cloacal sheath smooth; cloacal folds absent; vomerine odontophores present; choanae clearly visible; tongue approximately round, clearly notched and free posteriorly; pectoral fold absent; vocal slit extends from near base of tongue nearly to angle of lower jaw; vocal sac single, median, folds visible in gular region.

**Measurements of the holotype (mm).—**SVL 17.63; HL 5.31; HW 5.31; ED 2.15; TD 1.24; IOD 2.69; END 1.29; ELW 1.72; THL 8.87; TL 12.83; FT 7.16

**Coloration.—**The overall dorsal coloration is brown, with darker brown stains randomly dispersed on the dorsum and members. Several sparse black dots over the entire dorsum, including the dorsal surface of the hands, arms, feet, legs, and snout. Few sparse white granules on the dorsum. A well-marked white line from the posterior edge of the upper eyelid to the tip of the snout. White stripe on the tip of the snout, in the loreal region, below the eyes, and on the flanks. Gular region, chest, and belly cream-colored in ventral view. Finger and toe tips are bright orange. Dark red eye coloration. In preservative, the vivid brown coloration becomes faded and beige.

**Variation.—**Color variation is most related to the brightness and length of the white lines on the head and dorso-lateral region, which are always present but may vary from faint to well-marked, shape and size of the darker stains (Fig 3), and background color, which varies from light to dark brown. The sparse dark dots and white granules also vary in quantity. Variation in the measurements of the type series is provided in Table 2.

**Table 2:** Morphometric measurements (mm) of the type series of *Dendropsophus liliae* sp. nov. Type series measurements are presented as average ± standard deviation (range). SVL (snout-vent length), HL (head length), HW (head width), ED (eye diameter), TD (tympanum diameter), IOD (interorbital distance), END (eye-nostril distance), ELW (eyelid width), THL (thigh length), TL (tibia length), and FL (foot length).

Measurements	Holotype	Type series
	MZUFV20707	(all males; n = 15)
SVL	17.63	17.90 ± 0.98(16.04–19.50)
HL	5.31	5.54 ± 0.38 (5.00–6.27)
HW	5.31	5.76 ± 0.43 (5.05–6.33)
ED	2.15	2.38 ± 0.23 (2.02–2.73)
TD	1.24	1.14 ± 0.13 (0.95–1.45)
IOD	2.69	2.49 ± 0.27 (2.09–3.11)
END	1.29	1.43 ± 0.14 (1.25–1.69)
ELW	1.72	1.68 ± 0.20 (1.36–2.02)
THL	8.87	8.54 ± 0.50 (7.73–9.59)
TL	12.83	13.03 ± 0.60 (12.16–14.31)
FT	7.16	7.97 ± 0.55 (7.10–8.96)

**Fig 4:**
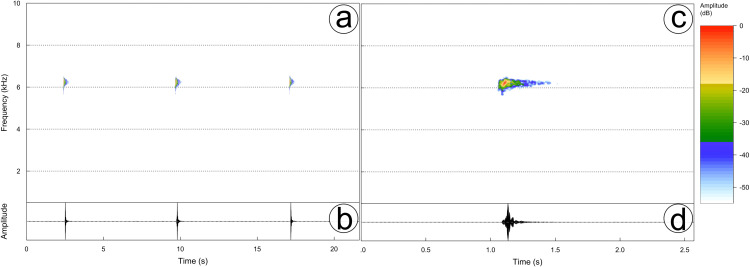
Advertisement call of *Dendropsophus liliae* sp. nov. (paratype MZUFV20679 from the type locality; air temperature 15.0ºC, water temperature 16ºC; SVL 19.09 mm). a) Oscillogram and b) spectrogram of a sequence of three advertisement calls, and c) oscillogram and d) spectrogram of one advertisement call.

**Advertisement call.—**Based on 21 calls from one paratype male (MZUFV20679) recorded at the type locality, the advertisement call of *D. liliae* sp. nov. consists of a single note (Fig 4) with duration of 0.065–0.200 s (0.103 ± 0.034 s) emitted at irregular intervals with a dominant frequency of 6029.3–6632.2 Hz (6395.4 ± 160.2 Hz). Males were calling about 1–3 m above the surface in a swamp inside a forest.

**Fig 5:**
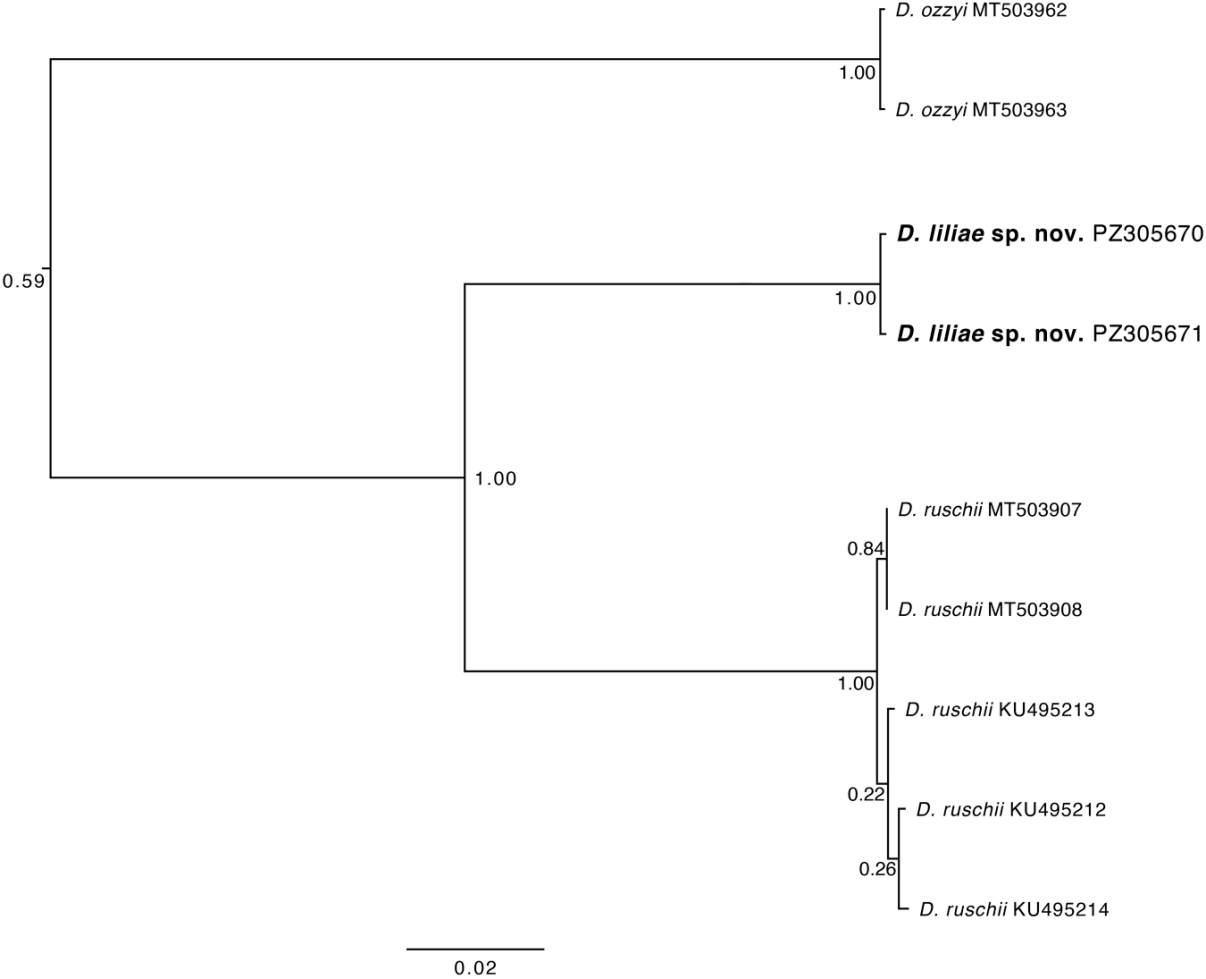
Bayesian phylogeny focused on the *Dendropsophus ruschii* species group and its closest relatives. This figure represents a section of the complete phylogeny presented in S1 Fig, based on the concatenated dataset of Orrico et al. (2021) with the inclusion of newly generated sequences of *Dendropsophus liliae* sp. nov. Numbers at nodes indicate Bayesian posterior probabilities. Nodes are labeled with the Bayesian posterior probability.

**Phylogenetic analysis.—**Average sequence divergence between *D. liliae* sp. nov. and the sampled within *D. ruschii* group ranges from 10.5% (*D. ruschii*) to 16.8% (*D. ozzyi*) (Table 3). In our phylogenetic analysis, *D. liliae* sp. nov. was robustly positioned within the *D. ruschii* group (pp = 1.0; Fig 5; S1 Fig) as the sister taxon of *D. ruschii* with strong support (pp = 1.0; Fig 5). Additionally, *D. ozzyi* was identified as the sister taxon of the clade formed by both *D. liliae* sp. nov. and *D. ruschii*, however with low support. Despite using a different analytical approach (Bayesian inference), we recovered a monophyletic *D. ruschii* group and an overall similar tree topology as Orrico et al. [18]. However, we focused on delimiting the new species (i.e., DNA barcoding) using 16S, which has proven to be a good marker for this purpose [37,38]. Nonetheless, genetic distance (10.5%), the close phylogenetic relationship, morphological resemblance, and geographic proximity confidently corroborate the affinities of *D. liliae* sp. nov. with *D. ruschii*.

**Table 3:** Uncorrected *p*-distances for a 373-bp aligned fragment of the mitochondrial 16S rRNA gene for individuals of the *Dendropsophus ruschii* group.

		1	2	3	4	5	6	7	8	9
1	*D. ozzyi* MT503962									
2	*D. ozzyi* MT503963	0.003								
3	*D. ruschii* MT503907	0.144	0.141							
4	*D. ruschii* MT503908	0.144	0.141	0.000						
5	*D. ruschii* KU495212	0.144	0.141	0.000	0.000					
6	*D. ruschii* KU495213	0.144	0.141	0.000	0.000	0.000				
7	*D. ruschii* KU495214	0.144	0.141	0.000	0.000	0.000	0.000			
8	*D. liliae* sp. nov. PZ305670	0.168	0.166	0.105	0.105	0.105	0.105	0.105		
9	*D. liliae* sp. nov. PZ305671	0.168	0.166	0.105	0.105	0.105	0.105	0.105	0.000	

**Fig 6:**
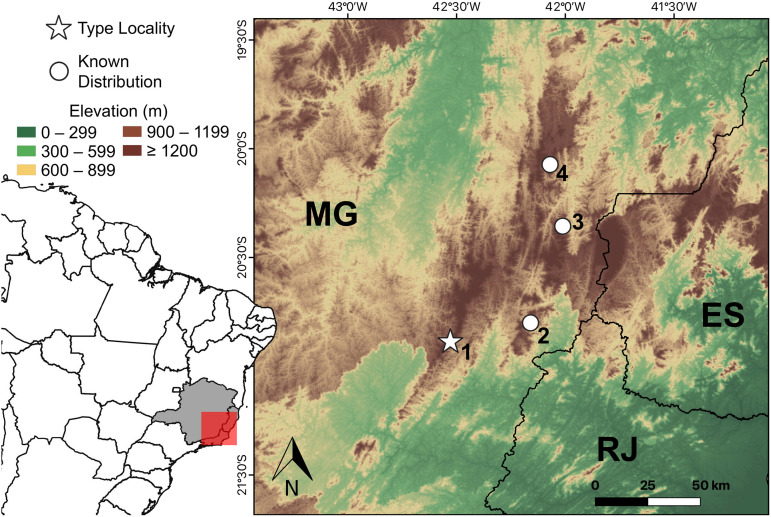
Geographic distribution of *Dendropsophus liliae* sp. nov. 1) Type locality, Serra do Brigadeiro, Ervália municipality, 2) Environmental Protection Area Pedra Dourada, Pedra Dourada municipality, 3) Parque Natural Municipal Sagui da Serra, Manhumirim municipality, 4) Private Natural Heritage Reserve Mata do Sossego, Simonésia municipality. State abbreviations: Minas Gerais (MG), Espírito Santo (ES), Rio de Janeiro (RJ). Localities are based on geographic coordinates obtained during fieldwork and are shown over a base map generated in QGIS using publicly available, public domain shapefiles. The map was produced by the authors and is published under a Creative Commons Attribution (CC BY 4.0) license.

**Distribution and natural history.—**Populations previously identified as *D. ruschii* along the northernmost portion of Serra da Mantiqueira in Minas Gerais [24,39] were reevaluated by us, and identified as *D. liliae* sp. nov. Therefore, *Dendropsophus liliae* sp. nov. is known from four localities in the northern section of the Mantiqueira Mountain Range, all in Minas Gerais (Fig 6). Across these localities, the species has been recorded at elevations ranging from approximately 1100–1270 m a.s.l. In its type locality, the area where we found the new species is characterized as a forest fragment with especially rich epiphytic flora mainly represented by Bromeliaceae and Orchidaceae. Serra do Brigadeiro is among the highest portion of a set of mountains comprising the northern part of the Mantiqueira Mountain Range, with a maximum of 1,985 m a.s.l., although the species appears to be restricted to habitats above 1000 m a.s.l. within this range, based on current records. The species was calling inside the forest, in a swampy area with emergent vegetation. There are few data on the biology or natural history of species of the *D. ruschii* group. The information we know is based on scarce information about their taxonomy and distributional records [18,20,21,39]. We collected and recorded the type series in November 2021 and November 2022, during weeks when it was raining. Males of at least three other species were calling in the same breeding sites used by *D. liliae* sp. nov. (*Aplastodiscus leucopygius*, *Scinax luizotavioi*, *Ischnocnema* aff. *parva*).

**Etymology.—** The specific epithet *liliae* is a patronym honoring Prof. Lília Maria Fraga Tostes (*in memoriam*) for her extensive contributions to biology education, her friendship, and her mentorship of DJS during his undergraduate studies. Prof. Lília, affectionately known as “Lilinha,” was a dear friend and a charismatic teacher widely known by the citizens of Muriaé, Minas Gerais (the hometown of DJS). Her kindness and charisma inspired many students to pursue careers in the biological sciences, and several of her former students are now professors who continue to carry her lessons forward.

## Discussion

We found *Dendropsophus liliae* sp. nov. is a member of the *D. ruschii* group and the sister taxon of the Atlantic Forest species, *D. ruschii*. The mitochondrial 16S gene is widely used and recommended as a DNA barcode for anurans [38,40], and has proved useful for delimiting and describing new species as part of an integrated taxonomic approach [41–43]. For speciose genera, such as *Dendropsophus* with over 100 species [44], comprehensive taxon sampling and genomic-scale data, or at least multiple loci, are imperative for resolving phylogenetic relationships. Additional sequence data from the new species and inclusion in future studies that build on the recent phylogeny for *Dendropsophus* [18] will certainly improve our understanding of the evolutionary history of species in the *D. ruschii* group.

The existence of historical connections between Amazon and Atlantic Forest lineages of anurans is well established [9,11,12], and the *D. ruschii* group was highlighted as an example when it was organized [18]. The description of *D. liliae* sp. nov. makes two known lineages within the *D. ruschii* group in the Atlantic Forest (*D. liliae* sp. nov. and *D. ruschii*) and one in the Amazon (*D. ozzyi*). Recent studies on the diversification of other species groups of *Dendropsophus* in Atlantic Forest and Amazon have revealed several potentially new species [45,46]. Further study of species in the *D. ruschii* group with extensive population sampling could also reveal additional new lineages.

The description of *Dendropsophus liliae* sp. nov. provides further evidence for the high level of micro-endemism in the northern Serra da Mantiqueira [23,24], representing the most inland record of this lineage within the Atlantic Forest. The substantial genetic divergence observed between *D. liliae* sp. nov. and *D. ruschii* (10.5% in the 16S gene) strongly supports its status as an independent evolutionary lineage. As the sister taxon of *D. ruschii*, the recognition of *D. liliae* sp. nov. also contributes to the ongoing discussion on the taxonomic arrangement of this lineage, particularly regarding its potential relationship with the *D. decipiens* group [19]. Although a comprehensive reassessment is beyond the scope of this study, some diagnostic characters of *D. liliae* sp. nov. may provide useful insights for future taxonomic revisions. Finally, the reassignment of populations from Ervália and Simonésia, previously identified as *D. ruschii* [24,39], highlights how the diversity of the Atlantic Forest remains underestimated when not evaluated under an integrative taxonomic framework.

## Appendix I

Specimens examined

**Acronyms.**—MZUFV = Museu de Zoologia João Moojen; ZUFMS-AMP = Coleção Zoológica da Universidade Federal de Mato Grosso do Sul; MBML = Museu de Biologia Mello Leitão; MNRJ = Museu Nacional do Rio de Janeiro.

***Dendropsophus ruschii*.—**Brazil, Espírito Santo, Domingos Martins, Pedra Azul: MBML6669 (female), MBML6775, MBML6776, MBML6670, MBML6677, MBML7871, MBML7872, MBML6778 (males).

***Dendropsophus liliae* sp. nov.—**Brazil, Minas Gerais, Ervália, Serra do Brigadeiro: MZUFV20707 (holotype, male), MZUFV20696, MZUFV20693, MZUFV20695, MZUFV20691, MZUFV20697, MZUFV20692, MZUFV20694, MZUFV20690, ZUFMS-AMP7816, MZUFV20679, MZUFV20680, MZUFV20681), MNRJ 94194 (ex-MZUFV20703), MNRJ 94195 (ex-MZUFV20704), MNRJ 94196 (ex-MZUFV20705) (all males, paratypes).

## Supporting information

S1 Fig Complete Bayesian phylogeny of *Dendropsophus* and outgroup taxa based on the concatenated dataset of Orrico et al. (2021) with the inclusion of the newly generated sequences of *Dendropsophus liliae* sp. nov. Numbers at nodes indicate Bayesian posterior probabilities. The complete tree is provided to show the placement of the new species within the genus, whereas Fig. 5 presents only the *D. ruschii* species group and its closest relatives.
(PDF)
