# Supplementary figures and images for: A new species of Dendropsophus (Anura, Hylidae) of the D. ruschii group from the Atlantic Forest in Serra da Mantiqueira, Minas Gerais, Brazil
Source: PLoS One. 2026 Jun 23;21(6):e0351087. doi: 10.1371/journal.pone.0351087 (PMC13289915; doi:10.1371/journal.pone.0351087)

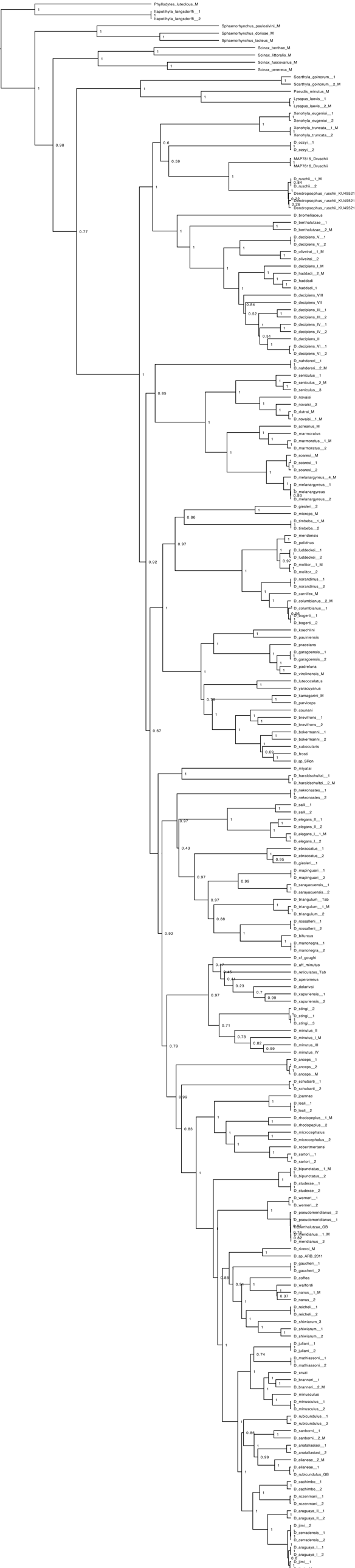

Supplement: S1 Fig — Numbers at nodes indicate Bayesian posterior probabilities. The complete tree is provided to show the placement of the new species within the genus, whereas Fig. 5 presents only the D. ruschii species group and its closest relatives. (PDF) [file pone.0351087.s001.pdf]
